# Importance of embryo aneuploidy screening in preimplantation genetic diagnosis for monogenic diseases using the karyomap gene chip

**DOI:** 10.1038/s41598-018-21094-6

**Published:** 2018-02-16

**Authors:** Gang Li, Wenbin Niu, Haixia Jin, Jiawei Xu, Wenyan Song, Yihong Guo, Yingchun Su, Yingpu Sun

**Affiliations:** Reproductive Medical Center, First Affiliated Hospital of Zhengzhou University, Zhengzhou, China

## Abstract

We investigated the incidence of aneuploidy in embryos from couples carrying monogenic diseases and the effect of embryo aneuploidy screening on the monogenic disease preimplantation genetic diagnosis (PGD). From November 2014 to April 2017, 36 couples carrying monogenic diseases were enrolled. The karyomap gene chip technique was used to analyze the blastocysts from the subjects and select normal embryos for transfer. A total of 43 single-gene PGD cycles were performed. A total of 687 eggs were obtained and 186 blastocysts were biopsed. After analysis via karyomap chip, 175 blastocysts received diagnostic results. In our monogenic disease PGD, 66.8% (117/175) of the embryos were diagnosed as normal or non-pathogenic (silent carriers), and 33.2% (58/175) of the embryos were diagnosed as abnormal or pathogenic. For preimplantation genetic screening (PGS), the aneuploidy rate of embryos was 22.9% (40/175). Among embryos diagnosed as normal for monogenic diseases, 26.5% (31/117) of the embryos were aneuploid and could not be transferred. Thus, approximately 1/4 of normal or non-pathogenic blastocysts diagnosed based on monogenic disease PGD were aneuploid, indicating the necessity and importance of embryo aneuploidy screening during PGD for monogenic diseases.

## Introduction

Monogenic diseases refer to diseases or pathological traits controlled by a single pair of alleles. Monogenic diseases can be divided into autosomal dominant genetic diseases, autosomal recessive genetic diseases, X-linked dominant genetic diseases, X-linked recessive genetic diseases, and Y-linked genetic diseases. There are nearly 7,000 human monogenic diseases. Therefore, preimplantation genetic diagnosis (PGD) of monogenic disease is a substantial challenge.

At present, monogenic disease PGD utilizes a variety of methods. The traditional method is to apply single-cell polymerase chain reaction (PCR) and conduct linkage analysis of the short tandem repeats (STRs) related to the pathogenic locus, which requires individualized STR design for each monogenic disease^1^. In addition, this method can be applied to monogenic disease PGD only after the verification of the pedigree. This process takes 3 to 4 months, and the cost is high.

Recently, genome-wide, high-throughput methods such as single-nucleotide polymorphism (SNP) microarray and multiple-parallel sequencing have been applied in clinical practice of monogenic disease PGD. Haplarithmisis based on SNPs has been introduced to perform whole-genome haplotyping and copy-number profiling of single cells, concurrently^2,3^. Furthermore, multiple-parallel sequencing has been utilized to detect monogenic diseases and chromosome abnormality, simultaneously^4^. Moreover, karyomap gene chip has been used for monogenic disease PGD to avoid monogenic diseases and chromosomal anomalies, simultaneously^5^. This gene chip is designed to focus on genome-wide SNPs and can simultaneously analyze nearly 300,000 SNP loci. It can be used for linkage analysis of SNPs within or near a particular pathogenic locus, without the necessity of individual design of STR loci. Therefore, the chip can be used for PGDs of different monogenic diseases. In addition, a whole-genome SNP chip can simultaneously obtain the results from genotyping and preimplantation genetic screening (PGS) for aneuploidy^6^. Therefore, the karyomap gene chip presents obvious advantages in monogenic disease PGD. The purpose of this study was to use the karyomap gene chip for monogenic disease PGD and PGS and to explore the incidence of aneuploidy in embryos from couples carrying monogenic diseases and the effect of embryo aneuploidy screening in monogenic disease PGD.

## Material and Methods

### Ethical approval and informed consent

All study methods were approved by Institutional Review Board and Ethics Committee of the First Affiliated Hospital of Zhengzhou University, and were performed in accordance with relevant guidelines and regulations. The informed consent was obtained from all subjects.

### Subjects

From November 2014 to April 2017, we enrolled 36 couples carrying monogenic diseases who sought fertility treatment from the Center for Reproductive Medicine, the First Affiliated Hospital of Zhengzhou University. A total of 43 cycles of PGD biopsy were performed, including 2 cycles of methylmalonic aciduria associated with homocysteinemia, 6 cycles of spinal muscular atrophy, 2 cycles of hereditary multiple exostoses, 1 cycle of Huntington’s chorea, 2 cycles of infantile polycystic kidney disease, 3 cycles of hemophilus A, 1 cycle of retinal pigment degeneration, 4 cycles of Duchenne muscular dystrophy, 1 cycle of X-linked adrenoleukodystrophy, 4 cycles of methylmalonic acidemia, 2 cycles of congenital insensitivity to pain with anhydrosis, 1 cycle of hereditary epilepsy, 1 cycle of Peutz-Jeghers syndrome, 1 cycle of phenylketonuria, 1 cycle of hereditary hypertrophic cardiomyopathy, 1 cycle of Alport syndrome, 1 cycle of spinocerebellar ataxia, 3 cycles of hepatolenticular degeneration, 1 cycle of congenital adrenal hyperplasia, 1 cycle of recessive congenital ichthyosis type 2, and 4 cycles of adult polycystic kidney disease.

### Ovarian stimulation, fertilization and Embryo culture

Controlled superovulation, *in vitro* fertilization, and embryo culture were conducted according to the routine procedures of our center. Patients were treated with the long program of the luteal phase. Eggs were collected 37 hours post-HCG and were fertilized via intracytoplasmic sperm injection (ICSI). Embryos underwent a sequential culture method and were maintained in a tri-gas incubator for the first 5–6 days, and the formed blastocysts then underwent blastocyst biopsy.

### Blastocyst biopsy and freezing

A hole was made with a laser in the zona pellucida of the embryo on day 3 of ICSI so that trophectoderm (TE) cells could herniate out of this hole. Herniated TE cells were subjected to biopsy on day 5 or 6 after ICSI. Three to 5 TE cells were aspirated with biopsy needles combined with laser-assisted cleavage. The TE cells were rinsed 3 times with phosphate-buffered saline (SAGE, USA) and were rapidly transferred to a 0.2-ml PCR tube for DNA amplification. After biopsy, the blastocysts were vitrified and stored in liquid nitrogen.

### Whole-genome amplification and karyomap chip detection

Whole-genome amplification (WGA) for TE cells obtained via biopsy was conducted using the QIAGEN REPLI-g Single Cell kit. The main steps were as follows: 4 μl of sample and 3 μl of buffer were mixed and incubated at 65 °C for 10 min, after which 3 μl of stop solution was added. Subsequently, each reaction system was supplemented with 40 μl of master mix, bringing the total volume of each reaction system to 50 μl, followed by incubation at 30 °C for 8 h, incubation at 65 °C for 3 min, and storage at 4 °C. The goal was to amplify genomic DNA at the picogram level by at least 10,000-fold to the nanogram level for the chip experiment. The second round of amplification for karyomap gene chip detection was conducted according to the karyomap chip manual. DNA was fragmented, precipitated, resuspended, hybridized, washed, extended, stained, and finally scanned, which took approximately 2 days. The chip was scanned using an Illumina HiScanSQ, and the karyomapping data were analyzed using Bluefuse software.

### Statistical analysis

Statistical analysis was performed using SPSS13.0 software. The proportional data were compared using Chi-squared analysis or Fishers Exact test, and the significant difference value was set at *P* < 0.05.

## Results

### General information

A total of 43 single-gene PGD cycles were performed for the 36 couples carrying monogenic diseases. The average age of the female subjects was 31.9 ± 4.1 years, and the average age of the male subjects was 32.4 ± 5.1 years. A total of 687 eggs were obtained, including 562 metaphase II (MII) eggs. All of these matured oocytes were subjected to ICSI. Blastocyst biopsies were conducted for a total of 186 blastocysts, including 137 D5 blastocysts and 49 D6 blastocysts (Table 1).

**Table 1:** General information of PGD for single gene disorder.

PGD for single gene disorder	
Number of cycle	43
Number of couple	36
Maternal average age (year)	31.9 ± 4.1
Paternal average age (year)	32.4 ± 5.1
Number of COCs	687
Number of MII oocytes (%)	562/687 (81.8%)
2PN (%)	505/562 (89.8%)
D3 good quality embryo (%)	330/505 (65.3%)
Biopsied blastocyst (%)	186/330 (56.3%)
D5 blastocyst (%)	137/ 186 (73.7%)
D6 blastocyst (%)	49/186 (26.3%)

This table demonstrates general information of PGD for single gene disorder including the number of harvested COCs, the number of biopsied blastocyst, ect.

### Rate of embryo aneuploidy in monogenic disease patients

**Figure 1:**
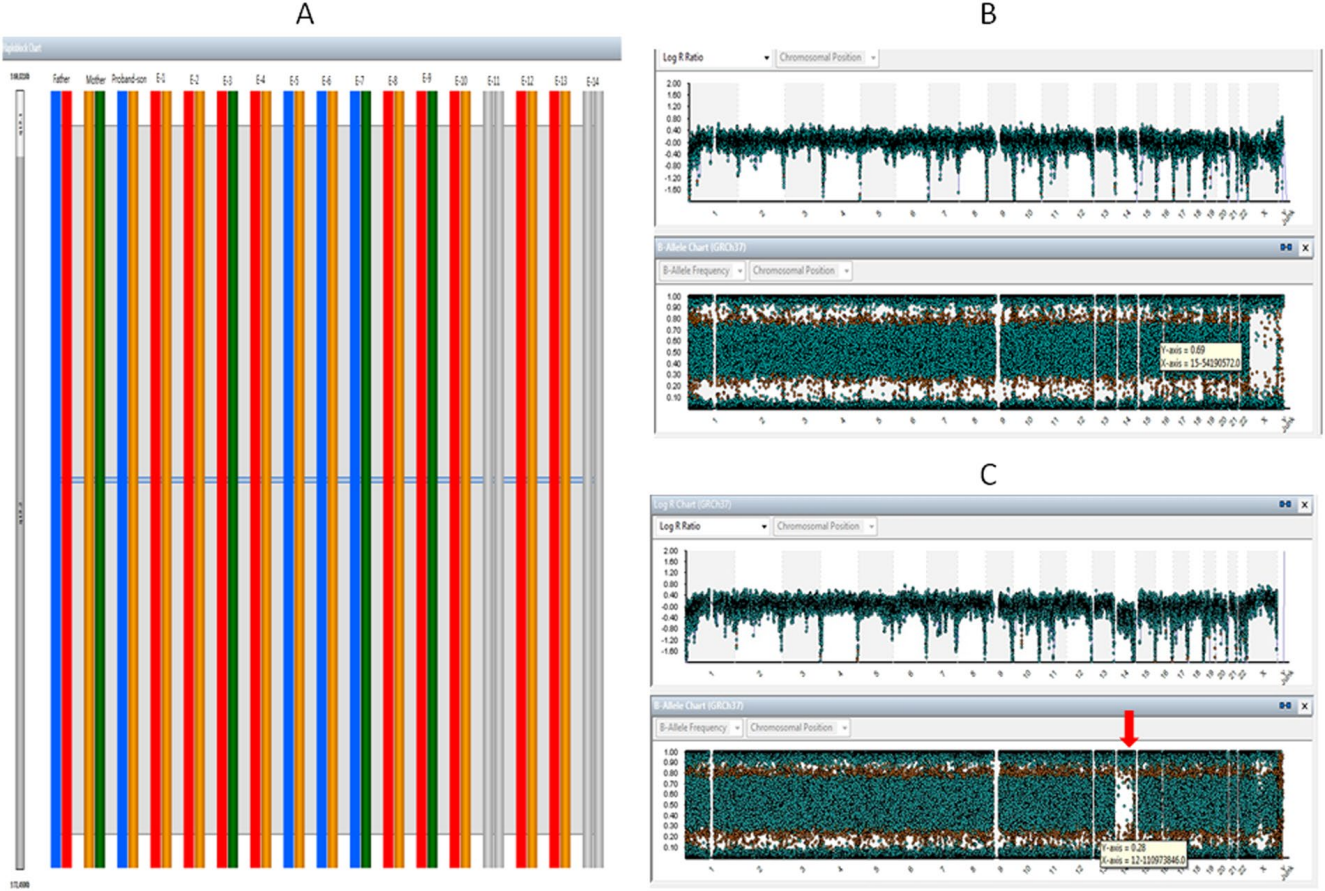
Haplotyping and karyotyping of preimplantation embryo using Karyomap SNP Microarray. This figure shows haplotyping and karyotyping of preimplantation embryo sample reading using Karyomap SNP microarrays. (**A**) Displays the haplotype outcome of Embryo-1(E-1)to Embryo-14(E-14) from a couple who is the Spinal Muscular Atrophy(SMA) carrier. The proband was the affected son with SMA. By haplotyping with BlueFuse Multi software analysis, the following is the diagnostic results for blasocyst. The diagnosis of E-1,2,4,7,810,12,13 is unaffected(carrier), E-3 and E-9 is unaffected(normal), E-5 and E-6 is affected. E-11and E-14 is amplification failure with no diagnosis. (**B**) Demonstrates the molecular karyotype of Embryo-1 with normal diploid diagnostic reading. Normal AA, AB and BB alleles and a 0 reading for the smooth log *R* ratio is observed for chromosome 1to 22 and one copy for X and Y. (**C**) Presents the abnormal molecular karyotype of Embryo-9 with a monosomy reading of chromosome 14, from a blastocyst stage embryo. AA and BB alleles are observed without AB alleles represented. A significant shift in the smooth log *R* ratio is observed, consistent with the monosomy karyotype for chromosome 14.

A total of 175 blastocysts received diagnostic results by karyomap chip. DNA amplification failed for 11 blastocysts, for a failure rate of 5.9%. For monogenic disease PGD, a total of 117 embryos were diagnosed as normal or non-pathogenic (silent carriers), and 58 embryos were diagnosed as abnormal or pathogenic. For PGS, the aneuploidy rate of embryos was 22.9% (40/175). Among embryos with a normal diagnosis for monogenic diseases, 26.5% (31/117) of embryos were aneuploid and could not be transferred (Tables 2,3, Fig. 1).

**Table 2:** Clinical results of PGD and PGS for single gene disorder.

Clinical results of PGD and PGS for single gene disorder	
Transferrable blastocysts (PGD + PGS)	86/175 (49.1%)
Non-transferrable blastocysts	89/175 (50.9%)
Due to PGD	49/89 (55%)
Due to PGS	31/89 (34.8%)
Due to PGD + PGS	9/89 (10.2%)
No results	11/186 (5.9%)
No. of frozen blastocyst transfer (FBT)	13
Clinical pregnancy of FBT	6/13 (46.1%)
No.of cycles waiting FBT	24
No. of cycles with no embryo available	6/43 (14%)

This table demonstrates clinical results of PGD and PGS for single gene disorder including the number of frozen blastocyst transfer (FBT), Clinical pregnancy rate of FBT, No. of cycles waiting FBT, No. of cycles with no embryo available, ect.

**Table 3:** Rate of embryo aneuploidy in monogenic disease patients.

Group	n	Euploidy n (%)	Aneuploidy n (%)
Unaffected embryos	117	86 (73.5)	31 (26.5)
Affected embryos	58	49 (84.5)	9 (15.5)
Total n (%)	175	135 (77.1)	40 (22.9)

This table illustrates the rate of embryo aneuploidy in the subgroup of unaffected and affected embryo diagnosed for monogenic disorder. Though there is no significant defference of aneuploidy rate between the two groups (P < 0.05 where * noted), among those embryos diagnosed as unaffected for monogenic diseases, 26.5% (31/117) of the embryos were aneuploid and could not be transferred.

Of the 43 PGD cycles, 6 cycles had no normal embryo for transfer, accounting for 13.9% of cycles. Frozen embryos were thawed and transferred for 13 cycles, and only 1 normal blastocyst was transferred in each cycle. A total of 6 cycles led to clinical pregnancy. Embryos from other cycles are still cryopreserved and waiting for transfer (Table 2).

## Discussion

### The advantages and disadvantages of the karyomap gene chip in monogenic disease PGD

Besides using traditional PCR technology, the monogenic PGD method has embraced high-throughput detection techniques, such as gene chip or multiple-parallel sequencing^4^. The traditional monogenic PGD is based on haplotype analysis using multiple STRs located inside or near the pathogenic gene, with the drawback of being time-consuming and laborious. These drawbacks can be addressed by the use of generic genome-wide haplotyping-based approaches such as SNP genotyping technology.

The karyomap gene chip is based on SNP genotyping techniques. The informative SNPs within 2 ~ 6 Mb from the pathogenic genes are selected to analyze the haplotype. The number of informative or key SNPs varies from a few to several tens, and as long as identified pathogenic genes can be provided, no patient-specific test is needed, which greatly reduces the time required for work-up prior to PGD. This monogenic PGD method makes use of indirect analysis with linked polymorphisms and provides high confidence with respect to the traditional single cell PCR which has rate of allele drop-out (ADO) about 10% and contamination with extraneous DNA. Problems such as ADO and contamination could lead to misdiagnosis^7,8^. This technology is applicable to all monogenic diseases and successful clinical applications have been reported^9,10^.

Another advantage of the karyomapping technology-based monogenic PGD is the capacity to simultaneously perform embryo aneuploidy screening^11,12^. The SNP gene chip is a more mature technology and has been successfully applied to chromosomal disease PGD and PGS. Therefore, the application of a karyomap gene chip can also be used for the diagnosis of embryo aneuploidy, thus avoiding abortions and adverse pregnancies caused by aneuploidy.

However, karyomapping technology still has drawbacks. Firstly, unlike direct single cell PCR analysis of interest gene for monogenic disorders, this technology requires pedigree information for haplotype analysis, and dominant diseases require three generations of pedigree analysis. The absence of clear pedigree information prevents linkage analysis and haplotype analysis. In addition, this technique cannot detect new mutations or the duplication of equivalent sequences. Therefore, it can only be used in PGD of monogenic diseases with unambiguous diagnosis, clear genetic patterns, and well-established pedigrees. Secondly, this technique also carries the risk that recombinations that occur within the alleles may cause uncertainty in the analysis results. In the event of recombination, sequences between adjacent SNP sites used in haplotype analysis may have base changes or sequence changes, leading to either misdiagnosis or missed diagnosis. However, this drawback is the consequence of biological processes of preimplantation embryos and may exist in all haplotyping analysis strategy for PGD. Moreover, as well as PGD using other methods, once pregnancies conceived with the assistance of this technique, prenatal diagnosis still is required to clarify the diagnosis.

### Necessity of performing embryo aneuploidy screening in monogenic disease PGD

Whether embryo aneuploidy screening (PGS) can improve clinical pregnancy rates and clinical outcomes has been controversial. Studies have shown that PGS1.0, which is characterized by blastomere biopsy and conventional genetic testing, such as fluorescence *in situ* hybridization (FISH) or PCR, not only fails to improve clinical outcomes but also reduces clinical pregnancy rates^13,14^. Thanks to the development of high-throughput whole-genome chromosome testing techniques, such as microarray and NGS, and the improvements of vitrification technology, PGS2.0, characterized by TE cell biopsy and high-throughput detection of all chromosomes, is considered by most people to improve clinical pregnancy rates, reduce abortions, and improve clinical pregnancy outcomes although the debate is still ongoing^15,16^.

In this study, we used TE cell biopsy and the karyomap SNP gene chip to analyze the whole-genome aneuploidy rate. The total aneuploidy rate of blastocysts was 22.9%. It should be noted that the average age of the females was 31.9 years. These results suggest that young couples can have a high proportion of aneuploidy in blastocysts-even culture of the embryo to a good quality blastocyst does not guarantee the absence of aneuploidy.

In this study, the rate of aneuploidy was 26.5% for blastocysts with normal or non-pathogenic diagnosis in monogenic disease PGD, demonstrating the necessity and importance of embryo aneuploidy screening in monogenic disease PGD. If the traditional monogenic disease PGD method had been applied, simultaneous embryo aneuploidy testing could not have been performed, and these embryos would have been transfered, leading to abortions or fetal malformations. These results also show that the application of the karyomap chip for PGS is practical.

As for PGS, the issue of embryonic mosaicism cannot be ignored although mosaic blastocysts were not found in our limited sample size study. Increasing data have been showing that mosaicism is not an exclusive characteristic of cleavage-stage embryos, but is also prominent at the blastocyst stage. The levels of mosaicism in cleavage-stage embryos range from 15% to 75% and blastocyst range from 3% to 24%. Moreover, 4–16% of blastocysts could be diagnosed as mosaicism using SNP microarray^17^.Up to now, there is still a lot of controversy on how to deal with mosaic embryos in the clinic, although the birth of healthy babies have been reported following the transfer of mosaic aneuploid embryos and this warrants more research^18^.

In summary, approximately 1/4 of blastocysts diagnosed as normal or non-pathogenic via monogenic disease PGD were aneuploid, indicating the necessity and importance of embryo aneuploidy screening and suggesting that embryo aneuploidy screening should be one of the important indicators for selecting embryos for transfer. Because SNP loci have wide coverage, the karyomap chip can be used in PGD for all monogenic diseases with clear diagnoses, unambiguous genetic patterns, and well-established pedigrees. The use of the karyomap SNP haplotype gene chip for monogenic disease PGD and PGS yields accurate results and has great potential in monogenic disease PGD and PGS^19^.

## Electronic supplementary material

Supplemental data
