# Supplementary material for: Importance of embryo aneuploidy screening in preimplantation genetic diagnosis for monogenic diseases using the karyomap gene chip
Source: Sci Rep. 2018 Feb 16;8:3139. doi: 10.1038/s41598-018-21094-6 (PMC5816636; doi:10.1038/s41598-018-21094-6)
Supplement: Supplementary file 1 — Supplemental data [file 41598_2018_21094_MOESM1_ESM.pdf]

Title: Importance of embryo aneuploidy screening in preimplantation genetic diagnosis for monogenic diseases using the karyomap gene chip

Authors: Gang Li, M.D., Ph.D., Wenbin Niu, Ph.D., Haixia Jin, Ph.D., Jiawei Xu, Ph.D., Wenyan Song, Ph.D., Yihong Guo, M.D., Ph.D., Yingchun Su, M.D., Yingpu Sun, M.D., Ph.D. (corresponding author)

Reproductive Medical Center, First Affiliated Hospital of Zhengzhou University, China

Correspondence: Yingpu Sun, M.D., Ph.D.

Address: 1# Jianshe Road, Zhengzhou, Henan Province, 450052, People's Republic of China.

Phone: +8613503841888;

Fax: +8637166913114;

Email: syp2008@vip.sina.com

Supplemental data of indication for PGD and diagnosis results of PGS abnormalities

Table 1   List of indications for which PGD by karyomapping has been applied so far.

Name of monogenic disorder	Hereditary mode	Cycles of PGD using karyomapping
methylmalonic aciduria associated with homocysteinemia	AR	2
spinal muscular atrophy	AR	6
hereditary multiple exostoses	AD	2
Huntington's chorea	AD	1
infantile polycystic kidney disease	AR	2
hemophilus A	XR	3
retinal pigment degeneration	AD	1
Duchenne muscular dystrophy	XR	4
X-linked adrenoleukodystrophy	XR	1
methylmalonic acidemia	AR	4
congenital insensitivity to pain with anhydrosis	AR	2
hereditary epilepsy	AD	1
Peutz-Jeghers syndrome	AD	1
phenylketonuria	AR	1
hereditary hypertrophic cardiomyopathy	AD	1
Alport syndrome	XD	1
spinocerebellar ataxia	AD	1
hepatolenticular degeneration	AR	3
congenital adrenal hyperplasia	AR	1
recessive congenital ichthyosis type 2	AR	1
adult polycystic kidney disease	AD	4

AR: Autosomal recessive, AD: Autosomal dominant, XR: X-linked recessive, XD: X-linked dominant.

Table 2 Embryos excluded from transfer because of PGS abnormalities (chromosomal anomalies)

family No.	monogenic disease	Gene/Locus of interest	PGD No.	blastocyst No.	blastocyst grade	PGS result
30849	methylmalonic aciduria associated with homocysteinemia	MMACHC	1	1	2BB	45,XX,-16
33336	spinal muscular atrophy	SMN1	4	2	2BB	46,XX,del(16)(q24.1→qter)
33336	spinal muscular atrophy	SMN1	4	3	2BB	45,XX,-1
33647	hereditary multiple exostoses	EXT1	5	1	3BB	46,XX(whole genome UPD）
33923	Huntington's chorea	HTT	6	1	3BB	45,XX,-8
34421	infantile polycystic kidney disease	PKHD1	8	1	3BB	43,XX,-6,-8,-18
35171	hemophilus A	F8	10	2	3BC	46,XX,del(4)(q28.3→qter)
35171	hemophilus A	F8	10	3	4BB	45,XY,-20
36458	retinal pigment degeneration	PRPF31	11	2	3AB	47,XY,+8
36458	retinal pigment degeneration	PRPF31	11	8	3BC	46,XX,del(20)(q12→qter)
36650	spinal muscular atrophy	SMN1	12	2	3BB	46,XX,del(4)(q31.21→qter)
37534	spinal muscular atrophy	SMN1	13	9	4BB	45,XX,-14
37534	spinal muscular atrophy	SMN1	13	12	6BB	45,XY,-7
33647	hereditary multiple exostoses	EXT1	15	1	2BB	46,XY,del(11)(p15.1→pter)
38432	Duchenne muscular dystrophy	DMD	16	1	3BC	46,XX,-2,-7,+11,+22
32134	spinal muscular atrophy	SMN1	17	1	2BC	45,XY,+13,-18,-22
32134	spinal muscular atrophy	SMN1	17	2	3BB	46,XX,Xq-
32134	spinal muscular atrophy	SMN1	17	5	2BB	45,XY,-22

39208	X-linked adrenoleukodystrophy	ABCD1	18	2	3BC	46,XX,1q-,Xq+
39208	X-linked adrenoleukodystrophy	ABCD1	18	5	2BB	45,XY,-16
40640	methylmalonic acidemia	MUT	21	4	3AB	46,XY,del(13)(q33.3→q34)
40640	methylmalonic acidemia	MUT	21	5	3AB	46,XX,dup(21)(q22.11→qter)
39493	hepatolenticular degeneration	ATP7B	22	1	2BB	45,XY,-8
39493	hepatolenticular degeneration	ATP7B	22	2	2BC	45,XX,-17
41043	adult polycystic kidney disease	PKD1	23	2	2BC	45,XY,-17
41043	adult polycystic kidney disease	PKD1	23	5	2BC	46,XX,1q-
42448	congenital insensitivity to pain with anhydrosis	NTRK1	26	5	6BB	46,XX,del(1)(q32.1→qter)
42448	congenital insensitivity to pain with anhydrosis	NTRK1	26	8	3BB	46,XY,dup(6)(q22.5→qter)
42432	Peutz-Jeghers syndrome	STK11	27	3	4BB	46,XY,dup(3)(q29→q24)
42432	Peutz-Jeghers syndrome	STK11	27	5	3BB	45,XY,-1
45053	Alport syndrome	COL4A	33	1	3BC	46,XY,del(17)(q21.32→qter)
45108	spinocerebellar ataxia	SCA2	34	7	3BC	46,XX,del(5)(p13.1→pter)
45415	recessive congenital ichthyosis type 2	ALOX12B	40	1	3BC	45,XY,-15
41043	adult polycystic kidney disease	PKD1	41	1	3BC	45,XY,-7
39493	hepatolenticular degeneration	ATP7B	43	1	3BC	45,XX,-18
46977	spinal muscular atrophy	SMN1	45	1	4BB	47,XY,+16
46977	spinal muscular atrophy	SMN1	45	2	3BB	45,XX,-2
46977	spinal muscular atrophy	SMN1	45	3	3BB	45,XY,-13

46977	spinal muscular atrophy	SMN1	45	4	3BC	45,XY,-15
46977	spinal muscular atrophy	SMN1	45	5	3BC	46,XX,del(17)(q25.1-q25.3)
